# Development and validation of a prognostic nomogram for rectal cancer patients who underwent surgical resection

**DOI:** 10.3389/pore.2023.1611014

**Published:** 2023-04-19

**Authors:** Bochao Zhao, Jingchao Wang, Zhicheng Ma, Haikun Ye, Tao Yang, Kewei Meng

**Affiliations:** Department of Gastrointestinal Surgery, Tianjin First Central Hospital, Tianjin, China

**Keywords:** survival, prognostic, nomogram, SEER, rectal cancer

## Abstract

**Objective:** The purpose of this study was to develop and validate a nomogram model for the prediction of survival outcome in rectal cancer patients who underwent surgical resection.

**Methods:** A total of 9,919 consecutive patients were retrospectively identified using the Surveillance, Epidemiology, and End Results (SEER) database. Significant prognostic factors were determined by the univariate and multivariate Cox analysis. The nomogram model for the prediction of cancer-specific survival (CSS) in rectal cancer patients were developed based on these prognostic variables, and its predictive power was assessed by the concordance index (C-index). Calibration curves were plotted to evaluate the associations between predicted probabilities and actual observations. The internal and external cohort were used to further validate the predictive performance of the prognostic nomogram.

**Results:** All patients from the SEER database were randomly split into a training cohort (*n* = 6,944) and an internal validation cohort (*n* = 2,975). The baseline characteristics of two cohorts was comparable. Independent prognostic factors were identified as age, pT stage, lymph node metastasis, serum CEA level, tumor size, differentiation type, perineural invasion, circumferential resection margin involvement and inadequate lymph node yield. In the training cohort, the C-index of the nomogram was 0.719 (95% CI: 0.696–0.742), which was significantly higher than that of the TNM staging system (C-index: 0.606, 95% CI: 0.583–0.629). The nomogram had a C-index of 0.726 (95% CI: 0.691–0.761) for the internal validation cohort, indicating a good predictive power. In addition, an independent cohort composed of 202 rectal cancer patients from our institution were enrolled as the external validation. Compared with the TNM staging system (C-index: 0.573, 95% CI: 0.492–0.654), the prognostic nomogram still showed a better predictive performance, with the C-index of 0.704 (95% CI: 0.626–0.782). Calibration plots showed a good consistency between predicted probability and the actual observation in the training and two validation cohorts.

**Conclusion:** The nomogram showed an excellent predictive ability for survival outcome of rectal cancer patients, and it might provide an accurate prognostic stratification and help clinicians determine individualized treatment strategies.

## Introduction

Colorectal cancer (CRC) is one of the most prevalent malignancies and a major cause of cancer-related mortality in both Eastern and Western populations (1, 2). Despite considerable advancements in cancer prevention and early detection, a high proportion of CRC patients were diagnosed at a locally advanced stage. To date, preoperative chemoradiotherapy and surgical resection with total mesorectal excision (TME) have become the standard treatment strategy for locally advanced rectal cancer patients (3–5). Multidisciplinary treatment modalities reduced the rate of locoregional recurrence, but overall survival of rectal cancer patients was still unsatisfactory (6, 7). Adjuvant chemotherapy following the curative resection is expected to improve the survival outcome of these patients, but a few evidence have shown that rectal cancer patients could not gain a survival benefit from adjuvant chemotherapy, particularly for those patients underwent preoperative chemoradiotherapy (8, 9).

The tumor, node, metastasis (TNM) classification of the American Joint Commission on Cancer (AJCC) is a widely used staging system for prognostic evaluation and treatment decision-making of rectal cancer patients. However, many well-established prognostic factors, such as serum carcinoembryonic antigen (CEA) level, differentiation type, lymphovascular invasion (LVI), perineural invasion (), circumferential resection margin (CRM), and the number of lymph node harvest, are not considered in the current staging system. Its predictive value for the survival outcome of rectal cancer patients might be limited in the precision medicine era. Therefore, appropriate prognostic stratification is warranted to determine who might benefit from the individualized treatment.

The nomogram is an intuitive graph of statistical model that generates a numerical probability of clinical event by incorporating important predictive factors. Recently, several nomogram models have been developed to predict the survival outcome of rectal cancer patients and shown an excellent prognostic performance (10–12). However, the development and validation of the nomogram models were limited by the sample size. In the present study, we identified independent prognostic factors for rectal cancer patients using a large population-based database, and develop a prognostic nomogram to help establish a risk stratification and make decision on therapeutic protocol.

## Materials and methods

### Patients and follow-up

All patients were retrospectively identified from the Surveillance, Epidemiology, and End Results (SEER) 18 Registries Research database (1973–2016). The eligibility criteria of enrolled individuals were as follows: (1). The surgical resection for primary rectal cancer was performed (2). The diagnosis of rectal cancer was confirmed by histopathology (3). No distant metastasis at presentation (3). No neoadjuvant chemotherapy and/or radiotherapy before surgery (4). Patient age ≥18 years old (5). The clinicopathological records were completed and follow-up duration was at least l month. Ultimately, a total of 9,919 consecutive patients who underwent surgical resection for rectal cancer between January 2010 and December 2015 were included in this analysis.

To test the predictive value of the prognostic nomogram, 202 rectal cancer patients who underwent curative resection in our institution from January 2015 and December 2019 were recruited as an independent cohort for the external validation. This study was approved by the Ethics Committee of Tianjin First Central Hospital (No. 2023000503), and was conducted under the principles of the Helsinki Declaration.

Demographic, clinicopathologic and survival data, including patient age, sex, race, year of diagnosis, preoperative carcinoembryonic antigen (CEA) level, pathological T category (pT stage), pathological N category (pN stage), pTNM stage, tumor size, differentiation type, histological classification, PNI, circumferential resection margin (CRM), lymph node yield, follow-up duration and vital status, were systematically collected and analyzed. The SEER database was accessed *via* free public website at www.seer.cancer.gov, and relevant data were extracted using the SEER*Stat software (version 8.3.6). Investigators received permission from the SEER program to access the original data.

The pTNM stage of rectal cancer patients was recorded according to the 7th edition of the TNM classification of the AJCC. In the current study cohort, the median period of follow-up was 33 months (range, 1–71 months). The primary outcome of survival analysis was cancer-specific survival (CSS), which was defined as the period from the date of diagnosis to the date of death owing to cancer-related causes. The observations of patients who died of other causes or were alive at the end of follow-up were defined as censored events.

### Statistical analysis

Categorical variables were reported as frequency distributions and were summarized in a descriptive table, and they were compared by Pearson’s chi-square test or Fisher’s exact test as appropriate. Continuous variables were expressed as median with range unless indicated otherwise and were compared using an unpaired Student’s t-test or Mann-Whitney test. The prognostic significance of clinicopathologic factors was assessed using the univariate Cox regression analysis, and the variables at a significant level (*p*-value <0.05) were candidates for the multivariate analysis. A Cox proportional hazard model with a backward stepwise selection procedure was used to determine independent prognostic factors for rectal cancer patients. Results for significant prognostic factors were presented as hazard ratio (HR) and its 95% confidence interval (CI).

Based on the regression coefficients estimated by the multivariate Cox analysis, the nomogram was established to predict the survival probabilities at 1-year, 3-year and 5-year. The predictive ability of the nomogram was assessed by calculating the concordance index (C-index), which estimates the probability of concordance between predicted and observed outcomes and is equivalent to the area under curve value of the receiver operating characteristic (ROC) (13). The value of the C-index should fall between 0.5 and 1.0. The maximum value of the C-index is 1.0, indicating a perfect prediction, whereas a C-index of 0.5 indicates that the nomogram model does not have sufficient predictive power (13). Calibration curves were plotted to evaluate the associations between predicted probabilities and the observed outcomes. In the calibration curve, the vertical axis represented the actual observations, whereas the horizontal one is the predicted probabilities. To reduce the overfit bias, the predictive performances of the nomogram were evaluated by bootstrapping method, in which the datasets were tested 1,000 times with random resampling each time. All statistical analyses were conducted using the statistical package for SPSS 22.0 (IBM Inc., New York, USA) and R software program with version 4.2.1 (http://www.r-project.org), and the statistical significance was accepted at a *p*-value <0.05.

## Results

### Patient characteristics

The entire cohort consisted of 5,924 males (59.7%) and 3,995 females (40.3%), and the proportion of patients aged 60 or older was 57.0% (5,657/9,919). Among 9,919 patients, 39.9% (3,962/9,919) had an elevated CEA level before surgery. The prevalence of lymph node metastasis in rectal cancer patients was 41.5% (4,116/9,919), and the median of metastatic lymph nodes was 2 (range, 1–36). The median of retrieved lymph nodes was 16 (range, 1–90), and 80.3% (7,969/9,919) of patients had at least 12 lymph nodes yield. Histologically, the proportion of well/moderately differentiated (WD/MD), poorly differentiated (PD) and undifferentiated (UD) adenocarcinoma were 86.8% (8,606/9,919), 10.9% (1,081/9,919) and 2.3% (232/9,919), respectively. According to the 7th edition of pTNM classification, the proportion of stage I, stage II and stage III patients were 17.9% (1,777/9,919), 40.6% (4,026/9,919) and 41.5% (4,116/9,919), respectively. The presence of PNI was detected in 1,272 patients (12.8%), and the incidence of CRM involvement was 16.7% (1,653/9,919) in rectal cancer patients.

In this analysis, 9,919 patients were randomly split into a training cohort (*n* = 6,944) and an internal validation cohort (*n* = 2,975) with a ratio of 7:3. Clinicopathologic characteristics of the two cohorts were summarized in Table1. The results indicated that the distributions of baseline characteristics between the training and validation cohort were comparable. The data split provided a balanced statistical power to develop and validate a prognostic nomogram for rectal cancer patients. Furthermore, 202 rectal cancer patients who underwent curative resection in our institution were recruited as an independent cohort for the external validation. The baseline characteristics of these patients were listed in the Table 2.

**TABLE 1:** Clinicopathological characteristics of rectal cancer patients in the training cohort and internal validation cohort.

Characteristics	Patients (%)	Patient cohort		
		Training (*n* = 6,944)	Internal validation (*n* = 2,975)	*p*-value
Age (years old)				0.433
<60	4,262 (43.0%)	2,966 (42.7%)	1,296 (43.6%)	
≥60	5,657 (57.0%)	3,978 (57.3%)	1,679 (56.4%)	
Sex				0.816
Female	3,995 (40.3%)	2,802 (38.3%)	1,193 (40.1%)	
Male	5,924 (59.7%)	4,142 (61.7%)	1782 (59.9%)	
Race				0.193
Black	766 (7.7%)	516 (7.4%)	250 (8.4%)	
White	7,933 (80.0%)	5,582 (80.4%)	2,351 (79.0%)	
Other	1,220 (12.3%)	846 (12.2%)	374 (12.6%)	
CEA level				0.571
Normal	5,957 (60.1%)	4,183 (60.2%)	1,774 (59.6%)	
Elevated	3,962 (39.9%)	2,761 (39.8%)	1,201 (40.4%)	
Differentiation type				0.448
WD/MD	8,606 (86.8%)	6,027 (86.8%)	2,579 (86.7%)	
PD	1,081 (10.9%)	747 (10.8%)	334 (11.2%)	
UD	232 (2.3%)	170 (2.4%)	62 (2.1%)	
Histological type				0.558
Adenocarcinoma	9,261 (93.4%)	6,490 (93.5%)	2,771 (93.1%)	
MUC/SRC	658 (6.6%)	454 (6.5%)	204 (6.9%)	
pT stage				0.104
T1-2	2,350 (23.7%)	1,686 (24.3%)	664 (22.3%)	
T3	6,526 (65.8%)	4,529 (65.2%)	1,997 (67.1%)	
T4	1,043 (10.5%)	729 (10.5%)	314 (10.6%)	
pN stage				0.117
N0	5,803 (58.7%)	4,123 (59.4%)	1,700 (57.1%)	
N1	2,823 (28.3%)	1,932 (27.8%)	871 (29.3%)	
N2	1,293 (13.0%)	889 (12.8%)	404 (13.6%)	
Tumor size (cm)				0.713
≤5	5,934 (59.8%)	4,146 (59.7%)	1,788 (60.1%)	
>5	3,985 (40.2%)	2,798 (40.3%)	1,187 (39.9%)	
CRM				0.804
Negative	8,266 (83.3%)	5,791 (83.4%)	2,475 (83.2%)	
Positive	1,653 (16.7%)	1,153 (16.6%)	500 (16.8%)	
Perineural invasion				0.922
No	8,647 (87.2%)	6,055 (87.2%)	2,592 (87.1%)	
Yes	1,272 (12.8%)	889 (12.8%)	383 (12.9%)	
Lymph node yield				0.549
<12	1,950 (19.7%)	1,376 (19.8%)	574 (19.3%)	
≥12	7,969 (80.3%)	5,568 (80.2%)	2,401 (80.7%)	

CEA: carcinoembryonic antigen; WD: well-differentiated; MD: moderately differentiated; PD: poorly differentiated; UD: undifferentiated; MUC: mucinous adenocarcinoma; SRC: signet-ring cell carcinoma; CRM: circumferential resection margin.

**TABLE 2:** Clinicopathological characteristics of 202 rectal cancer patients in the external validation cohort.

Characteristics	Patients (%)
Age (years old)	
<60	88 (43.6%)
≥60	114 (56.4%)
Sex	
Female	63 (31.2%)
Male	139 (68.8%)
CEA level	
Normal	143 (70.8%)
Elevated	59 (29.2%)
Differentiation type	
WD/MD	184 (91.1%)
PD	16 (7.9%)
UD	2 (1.0%)
pT stage	
T1-2	29 (14.4%)
T3	155 (76.7%)
T4	18 (8.9%)
pN stage	
N0	59 (29.2%)
N1	101 (50.0%)
N2	42 (20.8%)
Tumor size (cm)	
≤5	126 (62.4%)
>5	76 (37.6%)
CRM	
Negative	180 (89.1%)
Positive	22 (10.9%)
Perineural invasion	
No	145 (71.8%)
Yes	57 (28.2%)
Lymph node yield	
<12	24 (11.9%)
≥12	178 (88.1%)

CEA: carcinoembryonic antigen; WD: well-differentiated; MD: moderately differentiated; PD: poorly differentiated; UD: undifferentiated; CRM: circumferential resection margin.

### Univariate and multivariate analysis of prognostic factors for rectal cancer patients

The prognostic significance of clinicopathologic variables for rectal cancer patients in the training cohort was evaluated by the univariate and multivariate Cox analysis. In the univariate analysis, patient age (*p* < 0.001), race (*p* = 0.040), preoperative CEA level (*p* < 0.001), differentiation type (*p* < 0.001), histological classification (*p* < 0.001), tumor size (*p* < 0.001), pT stage (*p* < 0.001), pN stage (*p* < 0.001), the presence of PNI (*p* < 0.001), CRM involvement (*p* < 0.001) and lymph node yield (*p* = 0.043) were significantly associated with survival outcome of rectal cancer patients. After adjusting for potential covariates, age (*p* < 0.001), advanced pT stage (pT3 vs. pT1-T2 stage, *p* = 0.003; pT4 vs. pT1-T2 stage, *p* < 0.001), lymph node metastasis (pN1 vs. pN0 stage, *p* < 0.001; pN2 vs. pN0 stage, *p* < 0.001), elevated CEA level (*p* < 0.001), tumor size ≥5 cm (*p* = 0.007), PD or UD (*p* < 0.001), PNI (*p* < 0.001), CRM involvement (*p* < 0.001) and at least 12 lymph nodes yield (*p* = 0.001) were identified as independent prognostic factors (Table 3).

**TABLE 3:** Univariate and multivariate Cox regression analysis of prognostic factors for rectal cancer patients in the training cohort.

Factor	Univariate analysis		Multivariate analysis	
	HR (95% CI)	*p*-value	HR (95% CI)	*p*-value
Age (years old)				
<60	Reference		Reference	
≥60	2.015 (1.746–2.325)	**< 0.001**	2.236 (1.935–2.583)	**< 0.001**
Sex				
Female	Reference		-	
Male	1.094 (0.892–1.341)	0.389	-	
Race				
Black	Reference		Reference	
White	0.789 (0.630–0.989)	**0.040**	0.847 (0.675–1.063)	0.153
Other	0.766 (0.577–1.018)	0.066	0.805 (0.605–1.070)	0.135
CEA level				
Normal	Reference		Reference	
Elevated	1.720 (1.512–1.957)	**< 0.001**	1.358 (1.188–1.551)	**< 0.001**
Differentiation type				
WD/MD	Reference		Reference	
PD	1.976 (1.670–2.339)	**< 0.001**	1.341 (1.126–1.597)	**< 0.001**
UD	2.443 (1.802–3.312)	**< 0.001**	1.746 (1.283–2.375)	**< 0.001**
Histological type				
Adenocarcinoma	Reference		Reference	
MUC/SRC	2.081 (1.700–2.548)	**< 0.001**	1.711 (0.975–1.407)	0.092
pT stage				
T1-2	Reference		Reference	
T3	1.917 (1.586–2.316)	**< 0.001**	1.355 (1.111–1.654)	**0.003**
T4	4.033 (3.218–5.054)	**< 0.001**	2.027 (1.580–2.601)	**< 0.001**
pN stage				
N0	Reference		Reference	
N1	1.860 (1.603–2.158)	**< 0.001**	1.589 (1.362–1.854)	**< 0.001**
N2	3.042 (2.581–3.585)	**< 0.001**	2.371 (1.979–2.840)	**< 0.001**
Tumor size (cm)				
≤5	Reference		Reference	
>5	1.515 (1.332–1.724)	**< 0.001**	1.207 (1.054–1.382)	**0.007**
CRM				
Negative	Reference		Reference	
Positive	2.292 (1.993–2.636)	**< 0.001**	1.609 (1.387–1.866)	**< 0.001**
Perineural invasion				
No	Reference		Reference	
Yes	2.572 (2.211–2.992)	**< 0.001**	1.557 (1.321–1.835)	**< 0.001**
Lymph node yield				
<12	Reference		Reference	
≥12	0.857 (0.737–0.995)	**0.043**	0.771 (0.661–0.898)	**0.001**

The bold value represent a statistical significance (*p* < 0.05).

### Development and validation of the prognostic nomogram

**FIGURE 1:**
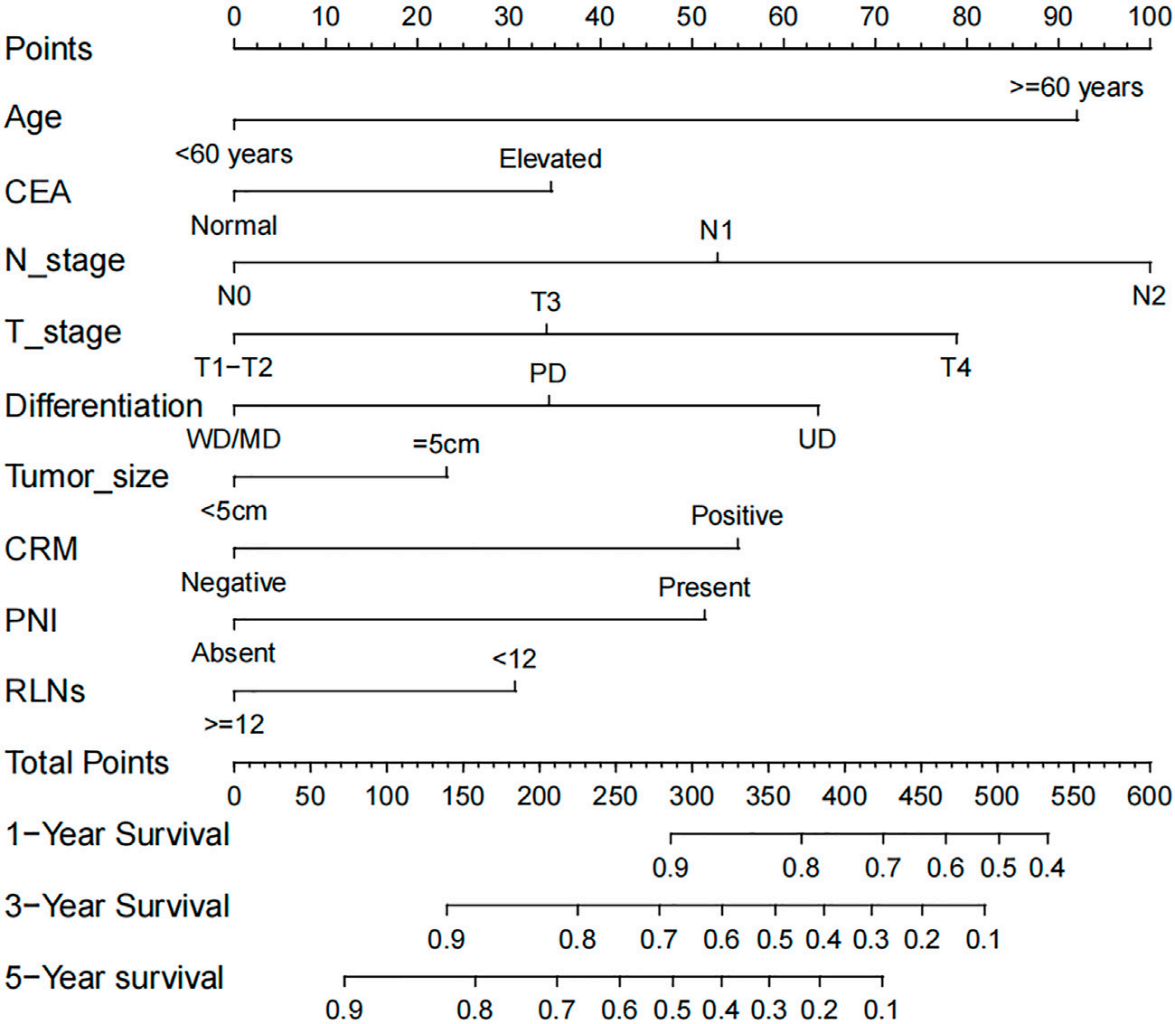
The prognostic nomogram of rectal cancer patients.

The nomogram was developed using identified 9 prognostic variables. As shown in Figure 1, the nomogram determined the survival probabilities of rectal cancer patients by assigning a score to each prognostic variable. The sum of these scores corresponds to the survival probability of patients. Higher total points in the nomogram, poorer survival for rectal cancer patients. In this predictive model, pN2 stage was the most important prognostic factor for rectal cancer patients followed by patient age and pT4 stage. pN1 and pN2 stage were assigned 53 points and 100 points, respectively. Similarly, patient age over 60 years old was assigned 92 points. pT3 and pT4 stage were assigned 34 points and 79 points, respectively. For example, a 55 years old patient with pT2N0 stage, elevated CEA level (for 35 points), tumor size of 7 cm (for 23 points), the presence of PNI (for 51 points), CRM involvement (for 55 points), undifferentiated type (for 64 points) and 10 lymph nodes harvest (for 31 points) had a total of 259 points. The predicted 3-year and 5-year CSS rates of this patient were 73.3% and 58.2%, respectively.

**FIGURE 2:**
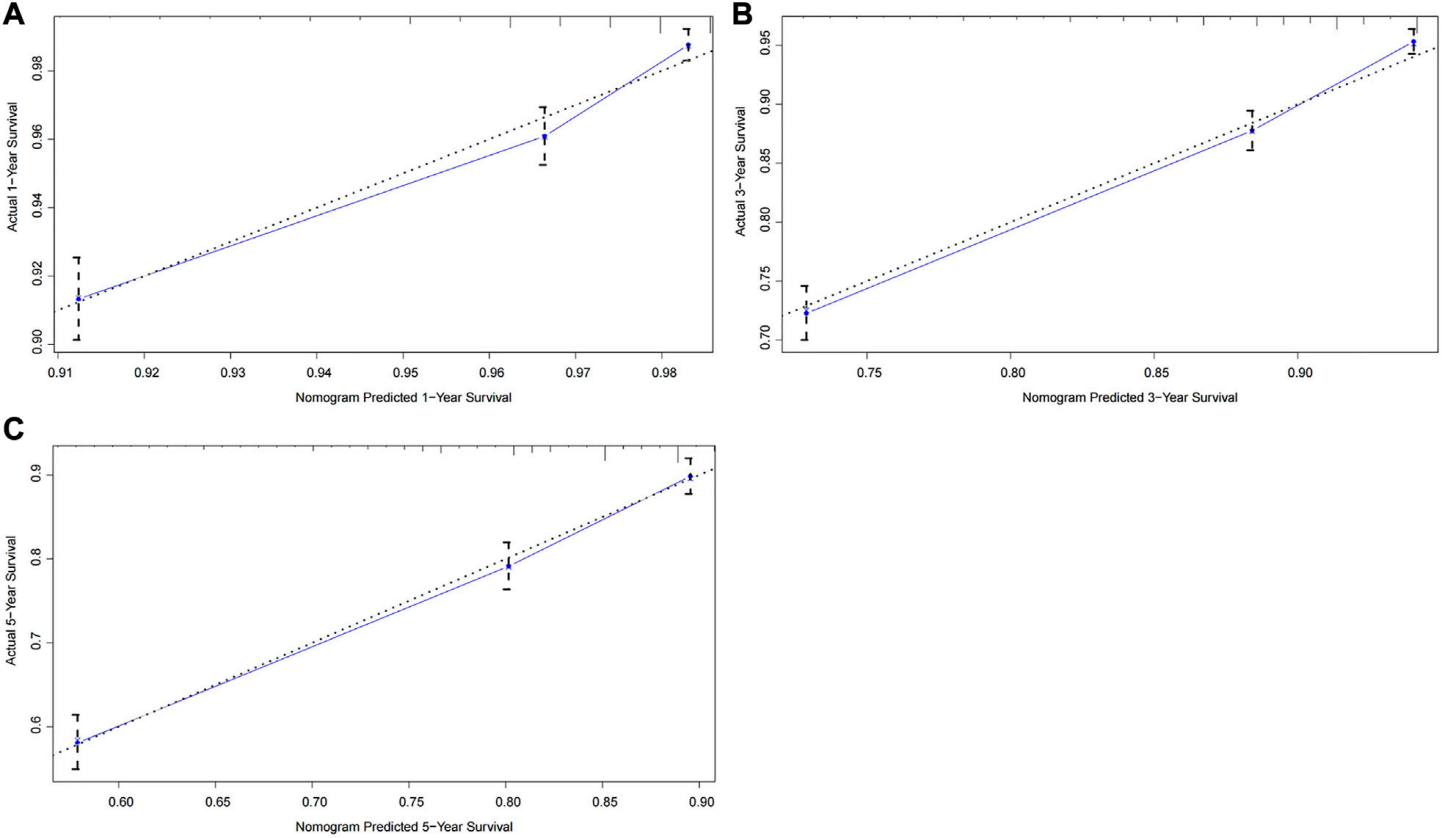
Calibration curves of the prognostic nomogram for the training cohort. **(A)** Predicted 1-year survival; **(B)** Predicted 3-year survival; **(C)** Predicted 5-year survival.

The C-index of the nomogram in the training cohort was 0.719 (95% CI: 0.696–0.742), which was significantly higher than that of the TNM staging system (C-index: 0.606, 95% CI: 0.583–0.629). Figure 2 illustrated calibration plots for the prediction of 1-year, 3-year and 5-year CSS in the training cohort. For a well-calibrated model, predicted outcomes should fall on a 45-degree diagonal line through the origin. As shwon in the calibration plots, a satisfactory conformance between the predicted and actual probability was observed (Figure 2). The nomogram had a C-index of 0.726 (95% CI: 0.691–0.761) for the internal validation cohort. There was no significant difference between the training and validation cohort for predictive performance, suggesting that the bias of model overfit was less evident. In addition, the nomogram had a higher C-index than the TNM staging system (C-index: 0.612, 95% CI: 0.577–0.647) for the prediction of survival outcome.

**FIGURE 3:**
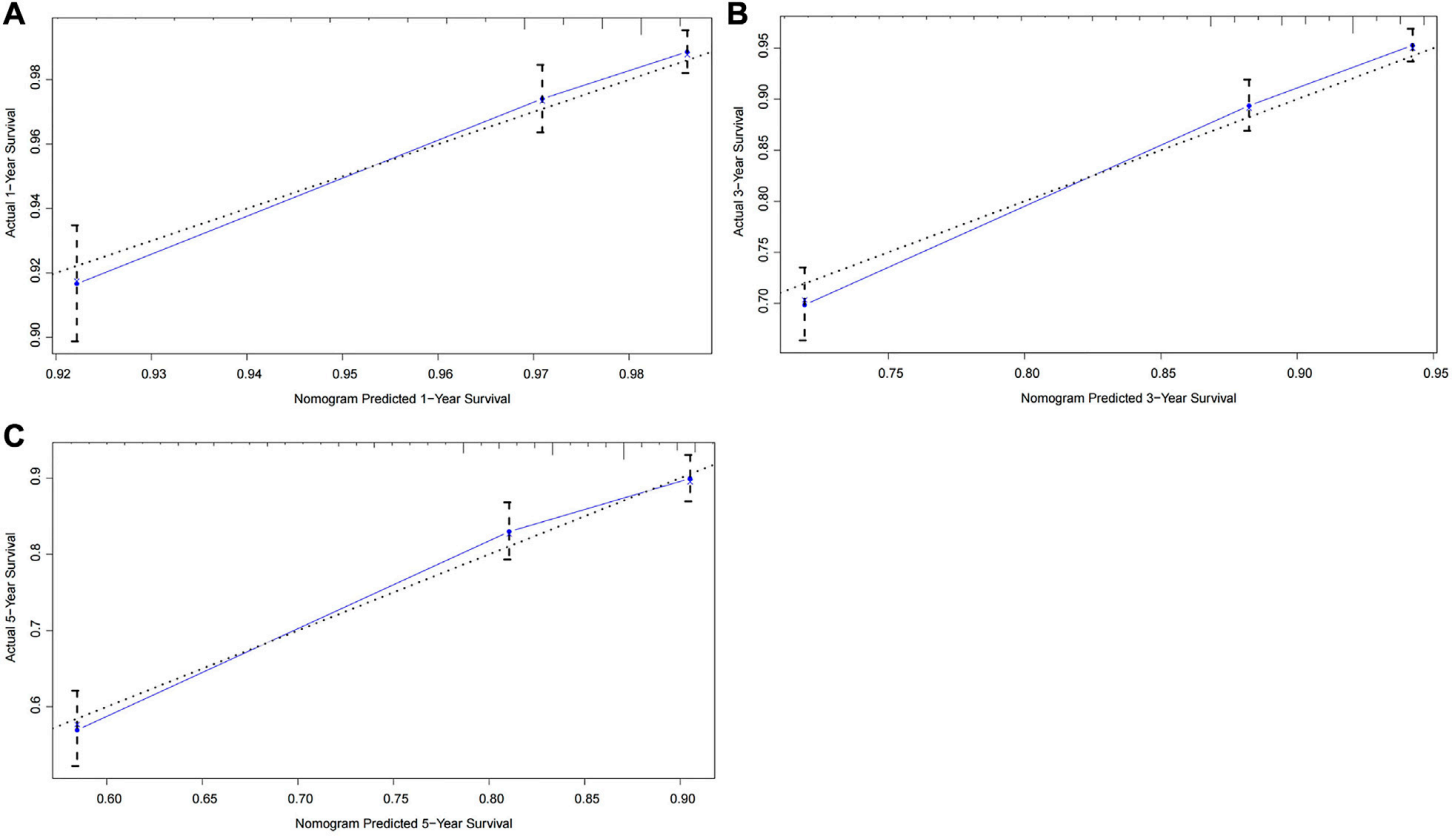
Calibration curves of the prognostic nomogram for the internal validation cohort. **(A)** Predicted 1-year survival; **(B)** Predicted 3-year survival; **(C)** Predicted 5-year survival.

**FIGURE 4:**
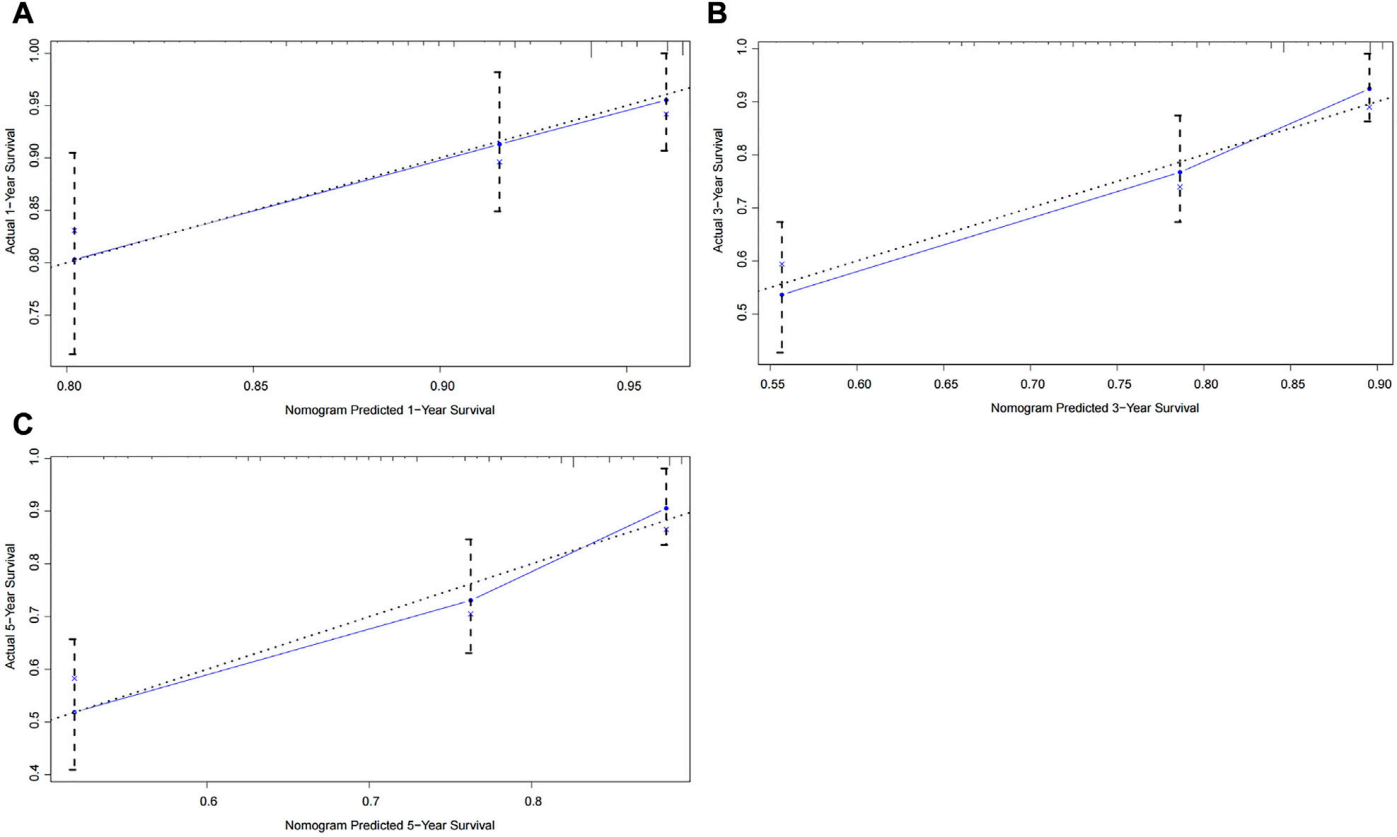
Calibration curves of the prognostic nomogram for the external validation cohort. **(A)** Predicted 1-year survival; **(B)** Predicted 3-year survival; **(C)** Predicted 5-year survival.

To further test the generalisability and clinical availability of the prognostic nomogram, an independent cohort composed of 202 rectal cancer patients were enrolled as the external validation. Compared with the TNM staging system (C-index: 0.573, 95% CI: 0.492–0.654), the prognostic nomogram still showed a better predictive performance, with the C-index of 0.704 (95% CI: 0.626–0.782). The calibration plots showed a good consistency between predicted probability and the actual observation in the internal and external validation cohort (Figures 3, 4).

## Discussion

Accurate risk stratification is crucial to prognostic assessment and subsequent treatment decisions for rectal cancer patients. In the precision medicine era, the single TNM staging system is insufficient to meet the therapeutic needs of all patients. Identifying as many prognostic variables as possible is helpful to determine individualized treatment strategies. In the present study, independent prognostic factors for rectal cancer patients were identified as age, pT stage, lymph node metastasis, elevated CEA level, tumor size, poorly differentiated/undifferentiated adenocarcinoma, PNI, CRM involvement, and inadequate lymph nodes yield, which have been reported in previous studies (14–20). Based on these data, we developed and validated a prognostic nomogram for rectal cancer patients. The results indicated that the nomogram had a higher C-index than the TNM staging system for the survival prediction (0.719 vs. 0.606), suggesting a better predictive ability. More importantly, its predictive performance was fruther validatd in an independent internal (C-index: 0.726 vs. 0.612) and external cohort (C-index: 0.704 vs. 0.573). These findings suggested that the nomogram model provided more accurate prognostic stratification for rectal cancer patients.

To date, there have been several nomogram models for survival prediction of rectal cancer patients. Using individual data from five large European randomized clinical trials, Valentini et al developed nomogram models that could predict local recurrence, distant metastasis and overall survival in locally advanced rectal cancer patients (10). After variable selection, patient age, pT stage, pN stage, surgical procedure, and adjuvant treatment were incorporated into the prognostic nomogram to evaluate the risk of recurrence and death. The C-index of the nomogram for the predictions of local recurrence, distant metastasis and overall survival were 0.68, 0.73 and 0.70, respectively (10). However, Shen et al reported that the predicted probabilities of relevant outcomes for a Chinese patient cohort could be overestimated by the nomogram model (21). The discrepancy may be interpreted as demographic heterogeneity and different treatment methods between Eastern and Western populations. On the other hand, the predictive performance of the nomogram for rectal cancer patients needs to be further optimized by introducing additional prognostic variables. Recently, Fan et al developed a prognostic nomogram incorporated clinicopathologic factors (patient age, pT stage, pN stage, differentiation type, PNI, tumor deposits) with serum biomarkers (CEA and CA19-9 level) to predict survival outcome of rectal cancer patients (12). The data indicated that the nomogram model had a better predictive performance than the TNM staging system (C-index: 0.71 vs. 0.58), and this finding was successfully validated by an external cohort (C-index: 0.69 vs. 0.57) (12). In the current study, several prognostic variables such as tumor size, CRM involvement and lymph node yield were further included in the nomogram model, and the results revealed a better predictive value. To avoid overfitting of the predictive model, it is essential to verify the generality of the nomogram. Using an internal cohort from the SEER database and an independent external cohort from our institution, the analytical results further supported the reliability and reproducibility of the prognostic nomogram.

Several limitations of this study require further discussion. Firstly, only those patients with complete clinicopathologic data were analyzed, which could introduce the potential selection bias. Secondly, adjuvant chemoradiotherapy and several well-established molecular variables such as microsatellite instability (MSI), KRAS and BRAF mutations were not included in this analysis since they were unavailable in the public SEER database. This is a major shortcoming of the current analysis. Thirdly, therapeutic protocols for rectal cancer patients have been evolved over the recent decade. Preoperative chemoradiotherapy followed by surgical resection with TME technique has become the standard treatment strategy for locally advanced rectal cancer (cT3/cT4 stage or lymph nodes involvement). However, the current analysis did not include patients who underwent neoadjuvant chemoradiotherapy, which limited the application of the nomogram for these individuals.

Despite the above-mentioned limitations, the prognostic nomogram was successfully developed by a large scale of real-world populations and was validated by an independent external cohort. Our data suggested that the nomogram model had an excellent predictive performance for the survival outcome of rectal cancer patients. It might provide an accurate prognostic stratification for rectal cancer patients and help clinicians make a decision on individualized treatment strategies.

## Data Availability

The supportive data of this manuscript are available from the Supplementary Material.
